# A network-based drug prioritization and combination analysis for the MEK5/ERK5 pathway in breast cancer

**DOI:** 10.1186/s13040-024-00357-1

**Published:** 2024-02-21

**Authors:** Regan Odongo, Asuman Demiroglu-Zergeroglu, Tunahan Çakır

**Affiliations:** 1 Department of Bioengineering, Faculty of Engineering, Gebze Technical University, Gebze, Kocaeli, 41400 Turkey; 2 Department of Molecular Biology & Genetics, Faculty of Science, Gebze Technical University, Gebze, Kocaeli, 41400 Turkey

**Keywords:** Network pharmacology, Transcriptome, Plant polyphenols, Breast cancer, MEK5/ERK5

## Abstract

**Background:**

Prioritizing candidate drugs based on genome-wide expression data is an emerging approach in systems pharmacology due to its holistic perspective for preclinical drug evaluation. In the current study, a network-based approach was proposed and applied to prioritize plant polyphenols and identify potential drug combinations in breast cancer. We focused on MEK5/ERK5 signalling pathway genes, a recently identified potential drug target in cancer with roles spanning major carcinogenesis processes.

**Results:**

By constructing and identifying perturbed protein–protein interaction networks for luminal A breast cancer, plant polyphenols and drugs from transcriptome data, we first demonstrated their systemic effects on the MEK5/ERK5 signalling pathway. Subsequently, we applied a pathway-specific network pharmacology pipeline to prioritize plant polyphenols and potential drug combinations for use in breast cancer. Our analysis prioritized genistein among plant polyphenols. Drug combination simulations predicted several FDA-approved drugs in breast cancer with well-established pharmacology as candidates for target network synergistic combination with genistein. This study also highlights the concept of target network enhancer drugs, with drugs previously not well characterised in breast cancer being prioritized for use in the MEK5/ERK5 pathway in breast cancer.

**Conclusion:**

This study proposes a computational framework for drug prioritization and combination with the MEK5/ERK5 signaling pathway in breast cancer. The method is flexible and provides the scientific community with a robust method that can be applied to other complex diseases.

**Supplementary Information:**

The online version contains supplementary material available at 10.1186/s13040-024-00357-1.

## Background

Complex diseases arise as a consequence of systemic alterations affecting several cellular processes [1]. In cancer, for instance, these alterations can be detected at several layers of the molecular organization, with changes in gene, protein, and metabolite expression levels being currently used to identify drug targets and disease biomarkers. Advances in high-throughput molecular profiling techniques such as transcriptomics have made it possible to collect genome-wide molecular data, and several studies in the literature have successfully used such data to link molecular changes to disease phenotype [2]. In particular, expressed genes/proteins in these diseases exist as an interconnected system forming molecular networks. These networks control specific cellular biological processes [1, 3, 4] and as such have provided a platform for discovering new drug targets, disease biomarkers and biological mechanisms underlying a disease condition. In the current era of precision medicine, such molecular networks have been leveraged to computationally prioritize drugs on disease target networks as well as identify potential drug pair combinations [1, 3, 5]. However, more refined computational pipelines are needed to make network-based approaches mainstream in drug research.

The field of network pharmacology applies network-based analysis techniques in drug research and has been shown to be more robust and accurate than traditional reductionist techniques in drug research [1, 3]. This approach evaluates drug candidates based on their systemic effects, i.e., the induced genome-wide molecular changes following a drug perturbation. In the literature, network proximity [4–6] and network biological function similarities [5, 6] are the main network metrics in common use. The former relies on the computation of topological distances between disease and drug target genes or proteins, while the latter is based on the semantic similarities between the altered biological processes between the nodes in the two networks. These metrics have been applied to prioritize or identify new uses for old drugs (i.e., drug repurposing) for complex diseases such as several cancers [4, 5], neurodegenerative diseases [7], cardiac diseases [8], metabolic syndrome [6] and viral infections [9, 10]. However, these approaches are limited by the simplistic view of the mechanism of drug action in that they do not take into consideration the directional consequences of a drug perturbation on its gene molecular targets. That is, a drug treatment can cause either an up- or downregulation of the target genes. This information is often disregarded by current studies employing network pharmacology. Methods that take the directionality metric into consideration are limited by a lack of robustness since they do not use a network-based approach [11]. Importantly, while there are network-based drug repurposing studies on cancers such as cervical cancer [12], there is no comprehensive study applying network proximity-based approach on breast cancer in the literature. Available studies on breast cancer have mapped transcriptome data on cellular pathways to identify disease targeted pathways and used network analysis to identify potential drug targets and multi-targets [13–15].

Accurate predictions from network pharmacology pipelines rely on comprehensively curated biological networks. In recent studies, integrating gene expression data (transcriptome) with protein–protein interaction networks to identify the active subnetwork characterizing a phenotype has gained prominence. There are several bioinformatic algorithms in the literature to accomplish this goal such as KeyPathwayMiner [16], and BioNet [17]. Context-specific subnetworks are enriched with important interconnected protein networks such as signaling and transcription regulatory networks that drive specific biological processes. Signaling networks propagate both intracellular and externally received information in a cell [18]. In cancer, the mitogen associated protein kinase (MAPK) signalling pathway drives carcinogenesis-related molecular processes such as cell proliferation, survival, apoptosis, and differentiation [19]. The MAP kinase 5—extracellular signal-regulated kinase 5 (MEK5-ERK5) pathway is a constituent of the MAPK pathway, and it has emerged as a potential carcinogenesis promoting signalling pathway in several cancers, including breast cancers, and is thus considered a promising target for therapeutic intervention [20–22]. Regrettably, there is no comprehensive study in the literature utilizing computational tools to identify potential drugs or drug combination candidates targeting the MEK5-ERK5 network.

In the current study, we used network pharmacology techniques to evaluate the activities of plant polyphenols on the MEK5/ERK5 signalling pathway in breast cancer. These compounds have received significant attention in the literature due to their potentials as anticancer drugs. Specifically, we propose an improved network pharmacology pipeline that leverages, in addition to target subnetworks and literature information, the transcriptional changes induced by these compounds in breast cancer. It uses techniques from network proximity, biological function similarity and transcriptional profile orthogonality. The proposed pipeline can, given a set of plant polyphenols and drugs with transcriptome profiles, perform (i) simulations to prioritise biologically highly active plant polyphenols capable of reversing the consequences of breast cancer pathology and (ii) plant polyphenol—drug combinations to prioritise combinations that have synergistic or enhancer properties on the disease target network. We demonstrate the consequences of the proposed computations and show their agreement with literature evidence.

## Methods

### Breast cancer transcriptome data

Breast cancer transcriptome data identified from a previous study were downloaded from the Gene Expression Omnibus (GEO) under accession number GSE42568 [23]. The dataset contains transcriptome profiles of 17 healthy controls, and 67 oestrogen receptor positive (ER +) patients generated using an Affymetrix microarray platform. First, we applied principal component analysis (PCA) to the dataset to identify potential outlier samples. For this dataset we did not find any outliers (Table 1 and Supplementary Fig. 1A-E). Then, healthy controls (*n* = 17) were compared with ER + breast cancer samples (*n* = 67) to identify differentially expressed genes.

**Table 1:** List of publicly available transcriptome datasets used in the study

Accession Number	Platform	Description	Number of samples
GSE42568	Affymetrix Array	Normal vs breast cancer gene expression data	Breast cancer: 104 (67 ER +) Normal: 17
GSE5200	Affymetrix Array	Control vs Genistein treated MCF-7 cell lines. 3µM and 10 µM Genisten	3 samples in each group
GSE25412	Affymetrix Array	Control vs Resveratrol treated MCF-7 cell lines. 150mM and 250 mM Resveratrol	3 samples in each group
GSE119552	Agilent Array	Control vs Apigenin treated MCF-7 cell lines. 10µM Apigenin	4 samples in each group
GSE23610	Affymetrix Array	Control vs Ferulic Acid treated MCF-7 cell lines. 0.1 and 1µM Ferulic Acid	3 samples in each group
GSE70138	Broad Institute Human L1000 epsilon	Control vs drug treated/genetically perturbed cell lines	960 drug perturbations

### Transcriptome data from plant polyphenol perturbation experiments

A GEO database search was performed to identify all available transcriptome profiles of luminal A cell models (MCF-7) exposed to plant polyphenols, with at least three samples per group. The MCF-7 cell line is a model of ER + and progesterone receptor positive (PR +) cancer cells and can be used to study the luminal A (LA) breast cancer subtype. It is known to be poorly aggressive, and noninvasive and has low metastatic capabilities. For the identified datasets, the PCA technique was used to check the quality of the datasets. Datasets with a clear separation of controls from plant polyphenol treated samples on a PCA plot were deemed to have the minimum quality requirements for use in this study (Table 1 and Supplementary Fig. 1). As a result, out of 20 datasets identified in the literature, we proceeded with four datasets, namely: GSE5200 (Genistein), GSE25412 (Resveratrol), GSE119552 (Apigenin) and GSE23610 (Ferulic acid).

### Differential gene expression analysis

The limma R package [24] was used to perform differential gene expression analysis. Briefly, a linear model was fitted to compare controls with disease/plant polyphenol treated samples. Gene log2 fold change (log2FC) and empirical Bayes *p*-values were then computed and used in subsequent analyses. Genes with FDR (false discovery rate) < 0.05 and a |log2FC|> 1.2, for upregulation or downregulation, were considered to be differentially expressed between the two conditions [25, 26]. We applied a |log2FC|> 1.2 in this analysis because standard log2FC thresholds such as 1.5 identified very few to no genes in some of the datasets used in this study.

### Drug signature data

Level 5 LINCS1000 Connectivity Map data were downloaded in GCTx format from GEO (GSE70138) to a local repository. Gene fold change data of the MCF-7 cell line exposed to 0.04, 0.12, 0.37 and 1.11 µM drug concentrations were then extracted using the signatureSearch R package [11]. We chose these doses since higher dosages are clinically difficult to administer and might also be cytotoxic. The dosage range also allowed us to select the most effective dose for each drug. For each of the drugs identified in the LINCS1000 database, we further checked whether they had any established gene targets in the literature. Specifically, we searched data from the Drug Gene Interaction database (DGIdb), accessed using the rDGIdb R package [27], and used evidence from the DrugBank [28] database to identify drugs whose targets have been experimentally characterised. Only drugs with available gene target information in the DGI database (*n* = *960*) were selected. The transcriptionally perturbed gene targets by these drugs were then identified based on a fold change cut-off of |log2FC|> 1.2 (i.e., for both up and down-regulated) after the exposure of the MCF-7 cell line to the drug. The identified genes were used to create a drug target subnetwork using the network contextualization approach described below. These subnetworks were subsequently used in the drug combination analysis.

### MEK5-ERK5 pathway and breast tissue related genes

The MAPK7 and MAP2K5 genes were separately used as search terms for MEK5 and ERK5 protein coding genes, and a set of 234 MEK5-ERK5- signaling pathway related genes were identified from the Human Integrated Protein–Protein Interaction rEference (HIPPIE) database. Likewise, 18 genes that are highly enriched in breast tissue relative to all other tissues in the human body were identified and downloaded from the Human Protein Atlas database [29]. These two gene lists were merged, forming a list with 252 genes (Extended Data Supplementary Table 1). This list was used in network contextualization analysis.

### Network contextualization

To generate context specific PPI networks, the walktrap algorithm [30] in the *cluster_walktrap* function of the igraph R package [31] was used to divide large networks into smaller and more specific modules in four steps. The Walktrap algorithm uses random walks to identify densely connected neighbourhoods in large networks. The random walks are used to calculate distances between nodes. Hierarchical clustering of the distances subsequently assigns nodes to different clusters. A cluster with relevance to a biological question can subsequently be identified from these clusters using pathway or gene ontology enrichment analysis techniques.

For all PPI networks and BioNet-derived subnetworks (see the sections below) used in this study, we applied this procedure and used Fisher’s exact test to compute the enrichment of the detected clusters in each network/subnetwork with the combined list of MEK5/ERK5 and breast tissue specific expressed genes (252 genes identified in the previous step). The cluster with the lowest *p*-value was selected for use in subsequent analysis.

### Protein–protein interaction network

The BioGRID (v4.4.219) human protein–protein-interaction (PPI) dataset was downloaded from the BioGRID repository (March 2023). Proteins with physical interactions detected in humans (Homo sapiens: 9606) were selected for use in subsequent analysis, which corresponded to 19,759 genes and 788,774 interactions. We further contextualised this network for breast tissue and MEK5-ERK5 pathway specificity using the walktrap algorithm (see the section above), resulting in a network with 7,511 genes and 198,057 interactions.

### Reconstruction of perturbed subnetworks for breast cancer and plant polyphenols

The BioNet R package [17] was used to integrate differential gene expression data (*p*-values) with the BioGRID PPI network contextualized in the preceding step to identify perturbed PPI subnetworks for breast cancer and for MCF-7 cell lines exposed to plant polyphenols. This tool scores the nodes of a background network using *p*-values from differential gene expression analysis to extract an active subnetwork. The FDR cut-off parameter in the BioNet algorithm was set at FDR = 0.05. With this approach, we identified subnetworks with genes and interactions, whose complete details are provided in Table 2. The identified subnetworks were further contextualised using the walktrap algorithm to make them more MEK5/ERK5-specific as described in the previous section. The network topological features of these contextualised networks are provided in Extended Data Supplementary Table 2.

**Table 2:** Details of the reconstructed protein–protein interaction subnetworks from transcriptome data. This table shows the number of active genes and interactions identified in each dataset

GEO accession	Plant polyphenol	Dosage	Number of genes	Number of interactions
GSE5200	Genistein	3 µM 10 µM	63 377	100 1,484
GSE25412	Resveratrol	150mM 250mM	1,523 730	11,276 3,606
GSE119552	Apigenin	10 µM	722	2,702
GSE23610	Ferulic acid	10µM	-	-

### Enrichment analysis

enrichR [32] was used for pathway enrichment analysis to investigate the mechanistic roles of a list of genes in MAPK-related processes. We checked for enriched gene ontologies based on biological processes (GO:BP), molecular function (GO:MF), and disease gene association network (DisGeNet). Subsequently, the Pathview R package [33] was used to map and visualize gene fold change values on the MAPK signalling pathway from the KEGG database. Given differential expression data, the PathView tool integrates expression values with a user-defined pathway network that is automatically parsed from the KEGG database and renders a graphical output that is scaled to show highly versus lowly expressed genes in the pathway.

### Network-based plant polyphenol prioritization

A network proximity approach [4, 6] was used to prioritize plant polyphenols on the MEK5/ERK5 contextualized human PPI network (the network with 7,511 genes and 198,057 interactions). The inputs to this analysis are the subnetwork of the breast cancer transcriptome and a subnetwork from a plant-polyphenol transcriptome (illustrated in Fig. 4A). As shown in Eq. 1 below, the proximity between the breast cancer subnetwork and plant polyphenol target subnetworks was computed using the network shortest distance method. This method first maps source and target nodes to a background network and then computes the sum of the minimum number of steps needed to move from a node in the source network to all the nodes in the target network. The sum of all distances is further normalized by the diameter of the background network.

1 $${d}_{\left({N}_{1},{N}_{2}\right)}=1-\frac{\frac{1}{\left|{N}_{1}\right|}\sum_{{n}_{1}\epsilon {N}_{1}}{min}_{{n}_{2}\in {N}_{2}}d({n}_{1},{n}_{2})}{Diam(network)}$$

In Eq. 1, *d* is the proximity score between the two networks, *N*_*1*_ and *N*_*2*_, hereby defined as the disease and plant polyphenol target subnetworks, respectively. *network* is the background network, hereby defined as the contextualized (specific to MEK5/ERK5 signalling pathway and breast tissue) human PPI network.

A second similarity score based on target gene ontology similarities between the two subnetworks was calculated using Wang’s method in the GOSemSim R package [34]. We refer to this similarity score as $NetSim$. This additional similarity layer provides a biological context for disease and drug target similarities.

A disease or drug perturbation may cause an increase in the expression patterns of some genes while decreasing the expression of other genes in the target subnetwork. For therapeutic applications, the objective of a drug treatment is to reverse the expression patterns of genes in the disease target subnetwork. Neither network proximity [4, 6] nor gene ontology similarity [6] captures this important phenomenon in biological networks. Thus, to address such cases, we extended the approach by Misselbeck et al. [6] for ranking drugs by considering a third score; orthogonality score ($orth score$). The $orth score$ was computed from the cosine similarity of fold changes (‘1’ for up-regulated and ‘-1’ for downregulated genes) of genes common to disease and drug target subnetworks. Since the cosine similarity score ranges from -1 to + 1 (for perfectly opposite and similar effects, respectively), drugs perturbing targets proximal to the disease target subnetwork but exert similar effects on such genes as the disease perturbation produces a lower score (close to -1), and vice-versa.

The final score was then defined as the summation of the three scores: network similarity score ($d$), gene ontology similarity score ($NetSim$), and orthogonality score ($orth score$), as given in Eq. 2 below:

2 $${Final\;Score}_{(N_1,N_2)}\;=\;d_{(N_1,N_2)\;}+{\;NetSim}_{(N_1,N_2)\;}+{\;orthscore}_{(N_1,N_2)}$$

Accounting for a drug’s orthogonal effect is not new as it is the approach used in the CMAP L1000 data for drug repurposing applications from differential gene expression signatures [11]. However, to our knowledge, it has not been applied before within a network context. From this approach, the Final Score can have a maximum value of 3 (i.e., out of 1 for network proximity, 1 for GO similarity scores and 1 for orthogonality score). This new scoring approach for ranking candidate drugs ensures that drugs with targets proximal to drug target subnetwork and exerting opposite effects on the disease target genes are ranked higher and vice-versa.

### Network-based plant polyphenol and drug combination analysis

We aimed to prioritize drugs with targets similar to or different from the targets of the plant polyphenols as combination therapeutic applications. Drugs with similar targets to plant polyphenol targets can be used synergistically with polyphenols while those with targets different from plant polyphenols can be spectrum enhancers (illustrated in Fig. 5A). Network proximity analysis was performed as described in the preceding section using Eq. 1. However, in this case, the breast cancer target subnetwork was used as the background network (1,875 genes and 18,904 interactions). Plant polyphenol and drug target subnetworks were used as the source and target networks (*N*_*1*_ and *N*_*2*_ in Eq. 1), respectively. The drug target subnetwork was defined as the combined list of genes with |log2FC|> 1.2 (for up- and down-regulated genes from the LINC1000 CMAP data) obtained after exposure of MCF-7 cell lines to a drug (*n* = 960) and the set of the corresponding drug’s known gene targets available in the literature (extracted from DGIdb and DrugBank databases). This approach is an improvement of the approach devised by Misselbeck et al. [6] and Güney et al. [4], both of which focus on gene subnetworks built around known drug targets as opposed to the global effects of drugs on gene expression. Likewise, we obtained overall scores (the sum of network proximity, GO term similarity and orthogonality, as explained in the preceding step) between the plant polyphenol and the drug target subnetworks. These scores were then used to define two sets of potential drug combinations: (i) synergistic combinations, and (ii) target network spectrum enhancers. Here, synergistic combinations are drug combinations with the lowest orthogonality score (i.e., the drug and the polyphenol should change the expression of common target genes in the same direction in terms of up/down regulation) and highest combined network proximity and GO term similarity score. Target network spectrum enhancer, on the other hand, are drug combinations with low/no orthogonality scores (since they do not have common targets) and hence the lowest overall score since they are expected to perturb different parts of the breast cancer subnetwork. For the two groups of drugs (target network synergistic or enhancer drugs), we further considered the number of gene targets deregulated by each; and only prioritized drugs whose gene targets had a comparatively high overlap (ranked based on the number of genes common to the drug and breast cancer target subnetworks) with the background network.

## Results

Transcriptome datasets of ER + breast cancer cell lines (MCF-7) that were exposed to plant polyphenols (genistein, resveratrol, ferulic acid, and apigenin) were used in this study for network-based drug prioritization and combination analysis of the MEK5-ERK5 pathway in breast cancer. The polyphenol compounds belong to different classes of plant polyphenols, with genistein and apigenin being flavonoids, while resveratrol and ferulic acid are stilbenoid and hydroxycinnamic acid respectively. In the recent past, these compounds have received considerable attention in the literature due to their potential use as anticancer drug candidates.

**Fig. 1:**
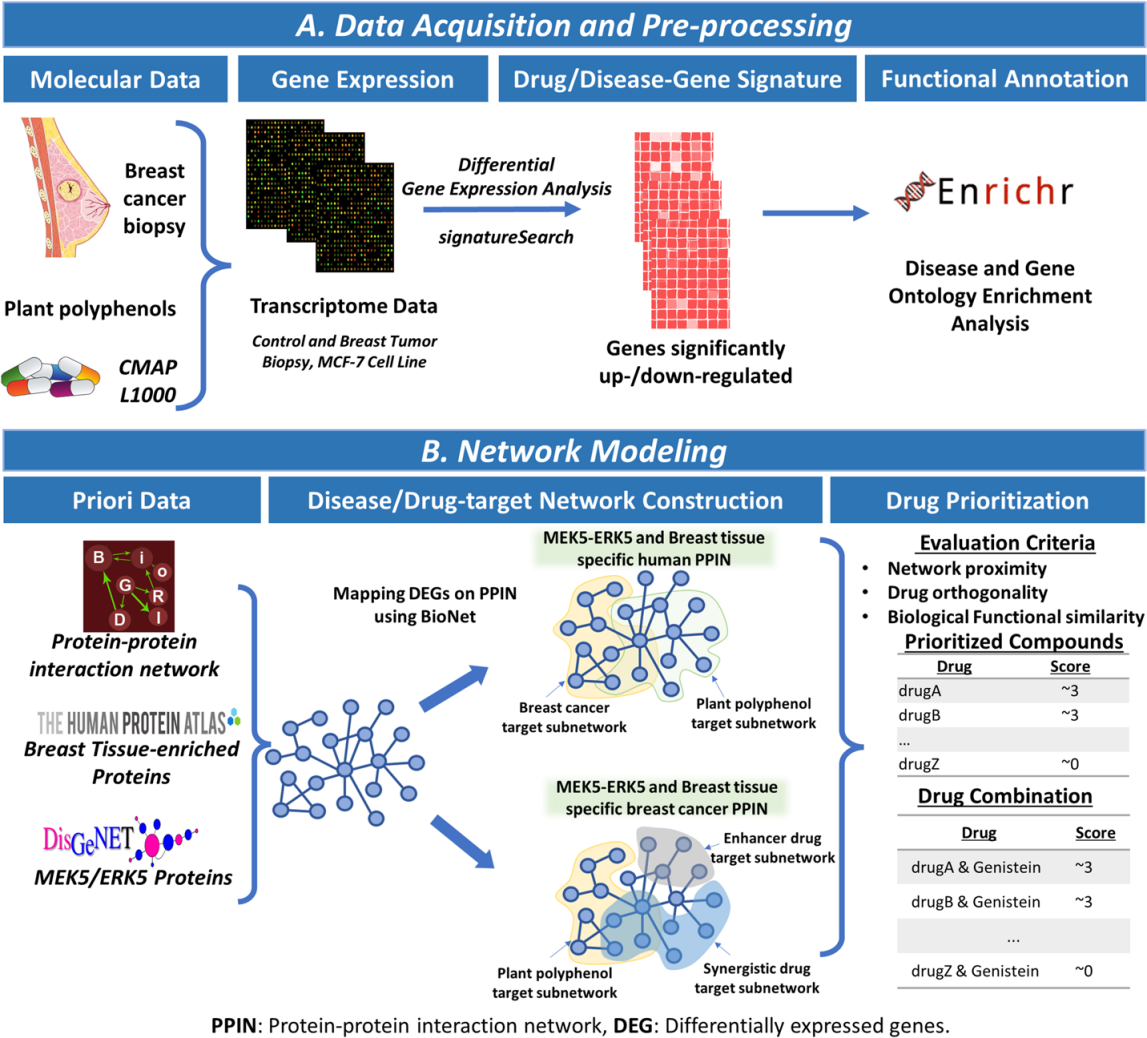
An illustration of the network-based pipeline for drug prioritization and combination analysis. The pipeline consists of two parts: **A**) Data acquisition from the literature and preprocessing to identify differentially expressed genes, and **B**) Network modelling illustrating the approach used to integrate differentially expressed genes from transcriptome data with the human protein–protein interaction network. These networks are then used as platforms to identify plant polyphenols proximal to the disease target network and approved drugs proximal to the plant polyphenol target network in the disease target network

Each transcriptome dataset was then mapped on the MEK5-ERK5 contextualized human PPIN to identify subnetworks with specific enrichment for the MEK5-ERK5 signaling pathway. Subnetworks are small networks derived from a larger network and tend to have a concise biological function. These subnetworks were subsequently used as inputs for the network-based drug prioritization of plant polyphenols and network-based combination with drugs. The pipeline (Fig. 1) computes the similarities between drug- and disease-target subnetworks using three different metrics: average shortest distance (network proximity), gene ontology (GO) term similarities, and orthogonality scores (Eq. 2). Drugs with high similarity scores were recommended as synergistic drugs for polyphenols whereas drugs with low similarity scores were recommended as network enhancers.

### Reconstruction of perturbed protein–protein interaction networks from transcriptome data

**Fig. 2:**
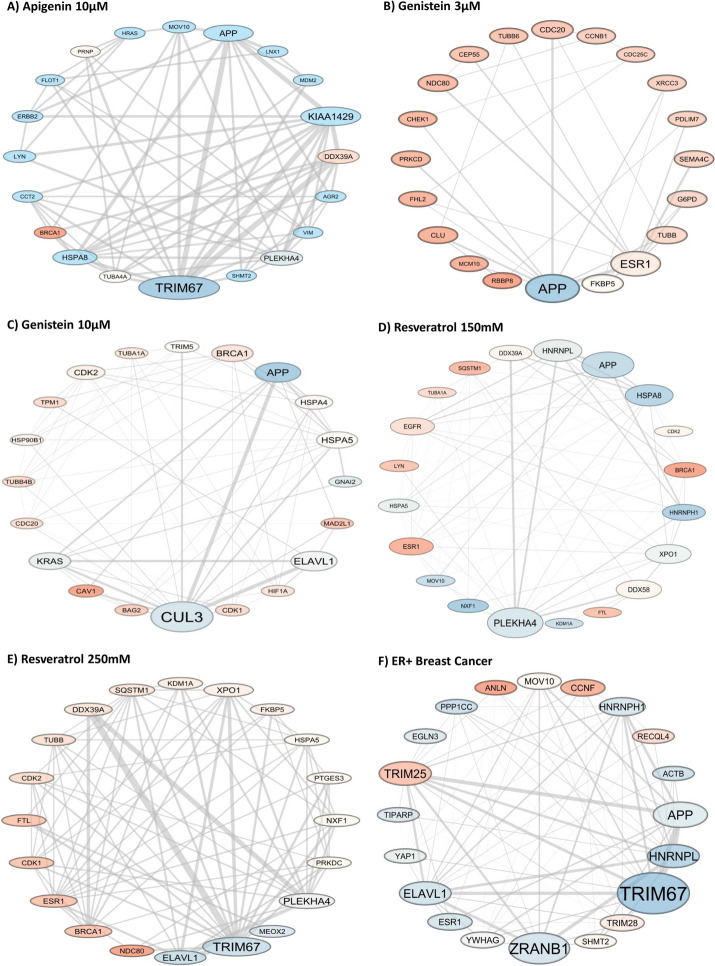
Subnetworks constructed in this study as visualized using Cytoscape. **A** Apigenin 10M, **B** Genistein 3M, **C** Genistein 10M, **D** Resveratrol 150mM, **E** Resveratrol 250mM, **F** ER + breast cancer. Only the first 20 genes with the highest betweenness centrality are shown for enhanced visualization

We first aimed to reconstruct and biologically characterising target subnetworks for (i) ER + breast cancer, (ii) plant polyphenols and (iii) drugs. We reconstructed the target subnetworks by mapping transcriptome data of plant polyphenols, ER + breast cancer and drugs on human MEK5-ERK5 specific PPIN using different strategies. For breast cancer and plant polyphenols, we mapped *p*-values from differential gene expression analysis. We identified target subnetworks for Genistein (3µM: 63 nodes and 100 edges, 10µM: 377 nodes and 1,484 edges), Resveratrol (150mM: 1,523 nodes and 11,276 edges and 250mM: 730 nodes and 3,606 edges), Apigenin (10µM: 722 nodes and 2,702 edges) and ER + breast cancer (1,875 nodes and 18,904 edges). Based on betweenness centrality analysis, these subnetworks were also enriched with genes previously annotated in breast and several other cancers (Fig. 2A-E and Extended Data Supplementary Table 2).

For instance, the APP (amyloid-beta precursor protein), ESR1 (estrogen receptor 1), TUBB (beta tubulin), and CUL3 (culin-3) genes have established links to promoting cancer cell survival, adhesion, differentiation, migration, and resistance to therapy in breast cancer [35–42]. Cytoplasmic localization of ELAVL1 (embryonic lethal abnormal vision-like protein 1) and BRCA1 (breast cancer 1) gene mutations are associated with poor prognosis of breast cancer [43–46]. CDK2 (cyclin dependent kinase 2) gene regulates cell cycle progression and has been shown to influence CDK4/6-targeted inhibitor efficacy [47, 48]. Therefore, these subnetworks were able to prioritize cancer-related genes.

**Fig. 3:**
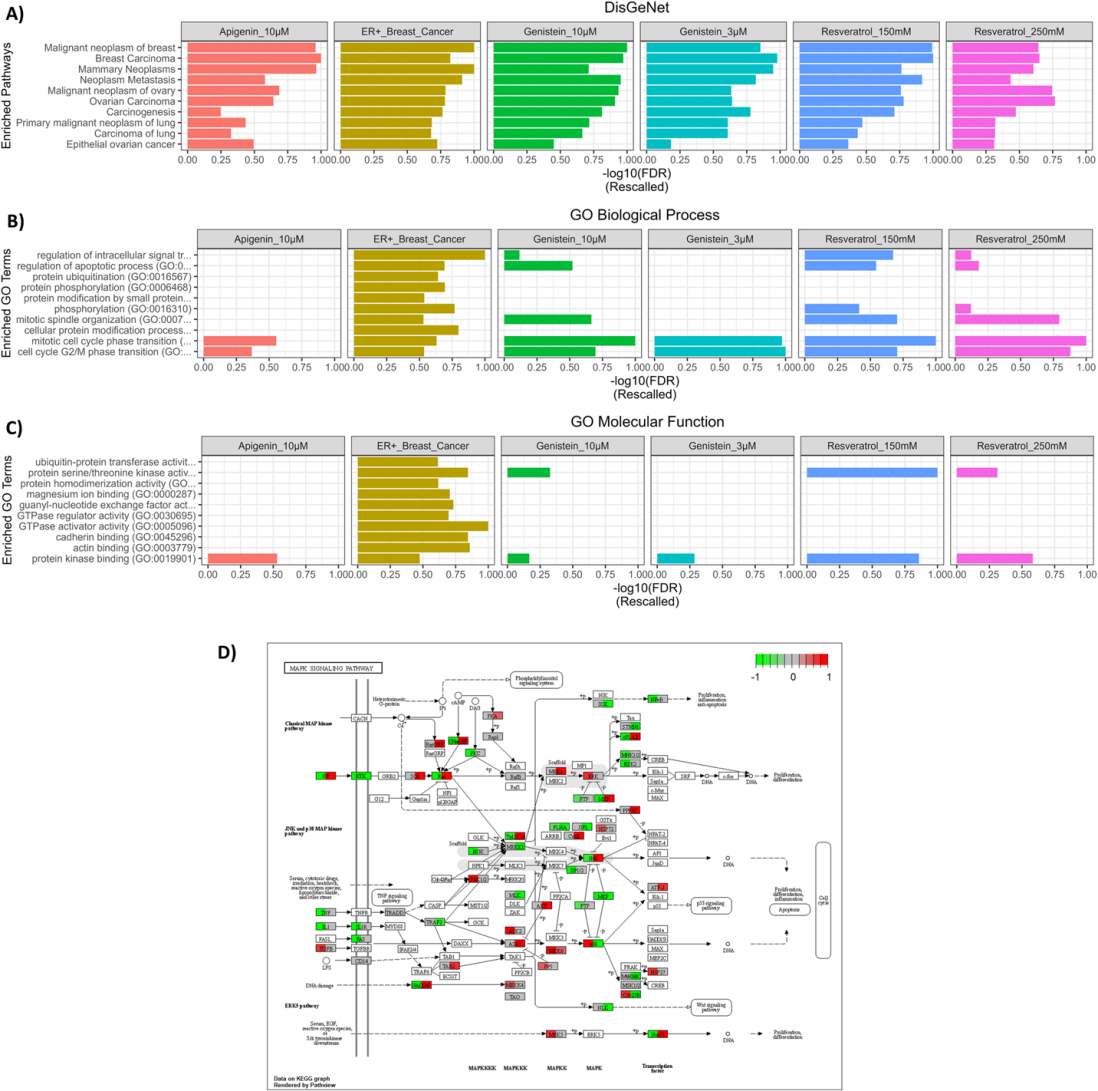
Enrichment analysis results. **A**-**C** Bar charts of significantly enriched terms from DisGeNet, GO Biological Process and GO Molecular Function in each dataset. The –log10(false discovery rate) was rescalled to between 0 and 1. DisGeNet: disease-gene network, GO: Gene Ontology. **D** Expression patterns of genes perturbed by Reseveratrol 150mM and ER + breast cancer in the MAPK pathway

In the enrichment analysis, all subnetworks were enriched with breast cancer related terms such as ‘Breast Carcinoma’, ‘Mammary Neoplasms’, ‘Malignant neoplasm of breast’ and malignancies in the DisGeNet database [49]*,* indicating the biological specificity of the identified subnetworks for breast tissue and breast cancer and a significant perturbation of protein networks in breast cancer and the ERK5/MEK5 signalling pathwa*y* (Fig. 3A and Extended Data Supplementary Table 3). From GO biological process enrichment analysis, we found ‘mitotic cell cycle’ as the commonly targeted biological process in all the datasets (Fig. 3B and Extended Data Supplementary Table 3). GO molecular function suggested protein kinase binding related processes as the main targeted process by all the perturbations – breast cancer and plant polyphenol exposure. This finding as well as the perturbation of several other kinases including mitogen activated kinase significantly support perturbation of signal transduction pathways in the constructed subnetworks(Fig. 3C and Extended Data Supplementary Table 3).

We next overlaid the gene fold changes from the target subnetworks by each plant polyphenol and breast cancer to visualize the differences and similarities between the effects of each perturbation on the MEK5-ERK5 pathway. While no gene mapped to the ERK5 protein, we found that MEK5 protein expression is deregulated by plant polyphenols but remains relatively unaffected in breast cancer. Similarly, Nur77 levels were high in breast cancer but were potentially reversed by Resveratrol (Fig. 3D). This protein is associated with cell death, immune response, and cell cycle and its high expression is indicative of poor prognosis in breast cancers [50]. The responses of MEK5/ERK5 obtained from other plant polyphenol compounds are shown in Supplementary Fig. 2A-D.

For each drug in the L1000 dataset whose significantly affected target genes were identified based on |log2FC|> 1.2 described in the methods, we constructed their target protein–protein interaction subnetworks. The topological properties of these subnetworks are provided in Extended Data Supplementary Table 4.

### Network-based plant polyphenol prioritization in breast cancer

We next evaluated the pharmacological effects of prioritized plant polyphenols on the MEK5-ERK5 signaling pathway subnetwork. First, we checked whether the subnetworks from plant polyphenols had direct targets in the breast cancer subnetwork. We found 175, 20, 110, 383 and 202 common nodes for Apigenin 10µM, Genistein 3µM, Genistein 10µM, Resveratrol 150mM and Resveratrol 250mM, respectively. These genes were mainly associated with ‘Cell cycle’, ‘p53 signaling pathway’,’ FoxO signaling pathway’, ‘regulation of mitotic cell cycle’,’ DNA replication’, and’regulation of apoptotic process’ among others (Extended Data Supplementary Table 5). The geodesic location of a gene in the target network can inform novel drug targets. We assessed the closeness and betweenness centrality of these genes. Ranked based on betweenness or closeness centralities, genes in the first 10 list with high betweenness also had high closeness centrality scores (23 genes in total). Genes such as APP, CDK1, CDK2, and ESR1 were the most frequently observed in this list and have been previously extensively characterized in breast cancer. Compared to average network topology centralities, most of the genes in this list had above average closeness centrality suggesting that most of the plant polyphenol gene targets can directly influence the majority of the genes in the breast cancer subnetwork (Extended Data Supplementary Table 6).

**Fig. 4:**
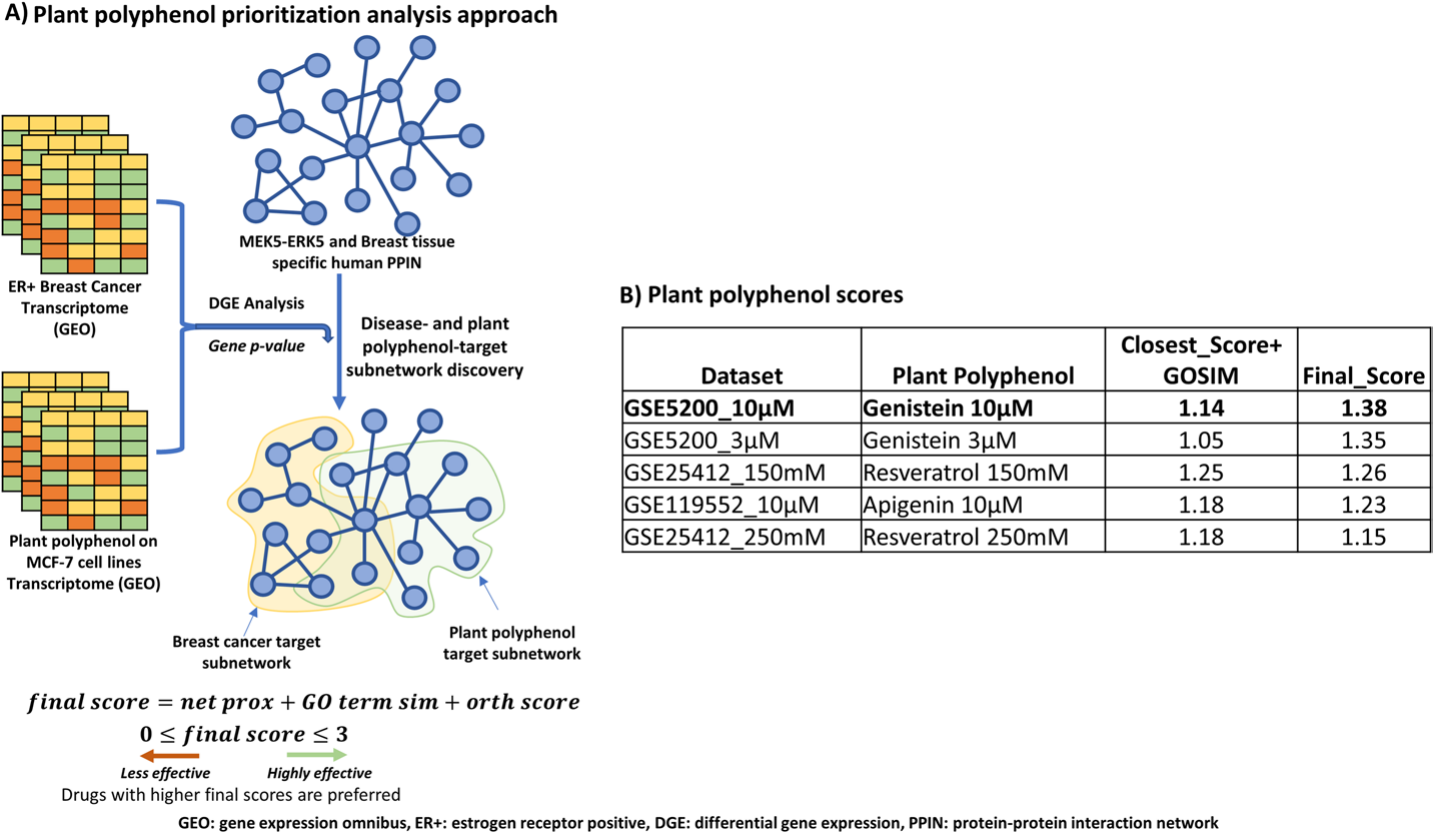
Network-based plant polyphenols prioritization analysis approach predicts Genistein 10µM as the most effective polyphenol in targeting the MEK5/ERK5 pathway in breast cancer. **A** A summary of the analysis strategy employed in this analysis showing the key steps. Plant polyphenols proximal to the disease target MEK5/ERK5 network are identified using network proximity, gene ontology similarity and target gene orthogonality analysis and are prioritized based on their corresponding final scores. **B** Plant polyphenols ranked based on the scores obtained by summing network proximity score + GO Term similarity (Closest_Score + GOSIM) and/or network proximity score + GO term similarity + orthogonality score (Final Score)

Next, using a combination of network proximity, GO Term similarity and orthogonality scores, we computed an overall score. When ranked based on $Network Proximity + GO Term similarity$, 150mM Resveratrol ($Score=1.25$) was the prioritized plant polyphenol. However, this changed when the orthogonality score was factored, with 10uM genistein ($Final Score= 1.38$) being prioritized (Fig. 4B) as the most effective plant polyphenol in targeting and reversing the effects of breast cancer on the MEK5/ERK5 target protein subnetwork. This observation indicates that the potential benefits from proximity + GO term similarity might be outweighed by a slight reversal of the breast cancer gene expression signature.

#### Network-based combination of drugs and plant polyphenols in breast cancer

We simulated the potential systematic effects of plant polyphenols and drug combinations on the MEK5-ERK5 pathway in breast cancer. Drug combination is a common therapeutic strategy used to increase the clinical potency of a given treatment regimen [51]. We identified drugs with synergistic potential and those with target network enhancer potentials as explained in the methods section. The latter group of drugs has not been previously extensively highlighted in the literature from a network pharmacology perspective. From each simulation, we first ranked the drugs based on their final scores and selected the first/last 50 for synergistic and enhancer drug categories respectively. Next, we considered the first/last 20 drugs in each group based on the number of common targets with the background network.

For Genistein 10µM, which we had previously prioritised among the plant polyphenols, we found dose-dependent differences in the set of drugs prioritized as potential combination candidates in the list of prioritized drugs. Importantly, among the predicted target network synergistic drugs, we found 8 drugs common to all doses, drugs such as fulvestrant [52], pralatrexate [53], dacinostat [54], camptothecin [55], indibulin [56], gemcitabine [57], daunorubicin [58], and epirubicin [59]. The majority of the drugs in this category, across all dosages, have been previously investigated and recommended for use as antineoplastics in breast and other cancers. Fulvestrant, for instance, is a strong ER inhibitor and is recommended for combination with cell cycle inhibitors in ER + breast cancer [52]. We had found a significant enrichment for cell cycle related terms by Genistein 10µM, indicating that this combination might exert greater effects by inhibiting cell proliferation. Likewise, dacinostat is a HDAC inhibitor that targets tumorigenesis processes such as angiogenesis and proliferation, suggesting that its combination with Genistein may exert a greater therapeutic effect on ER + breast cancer.

**Fig. 5:**
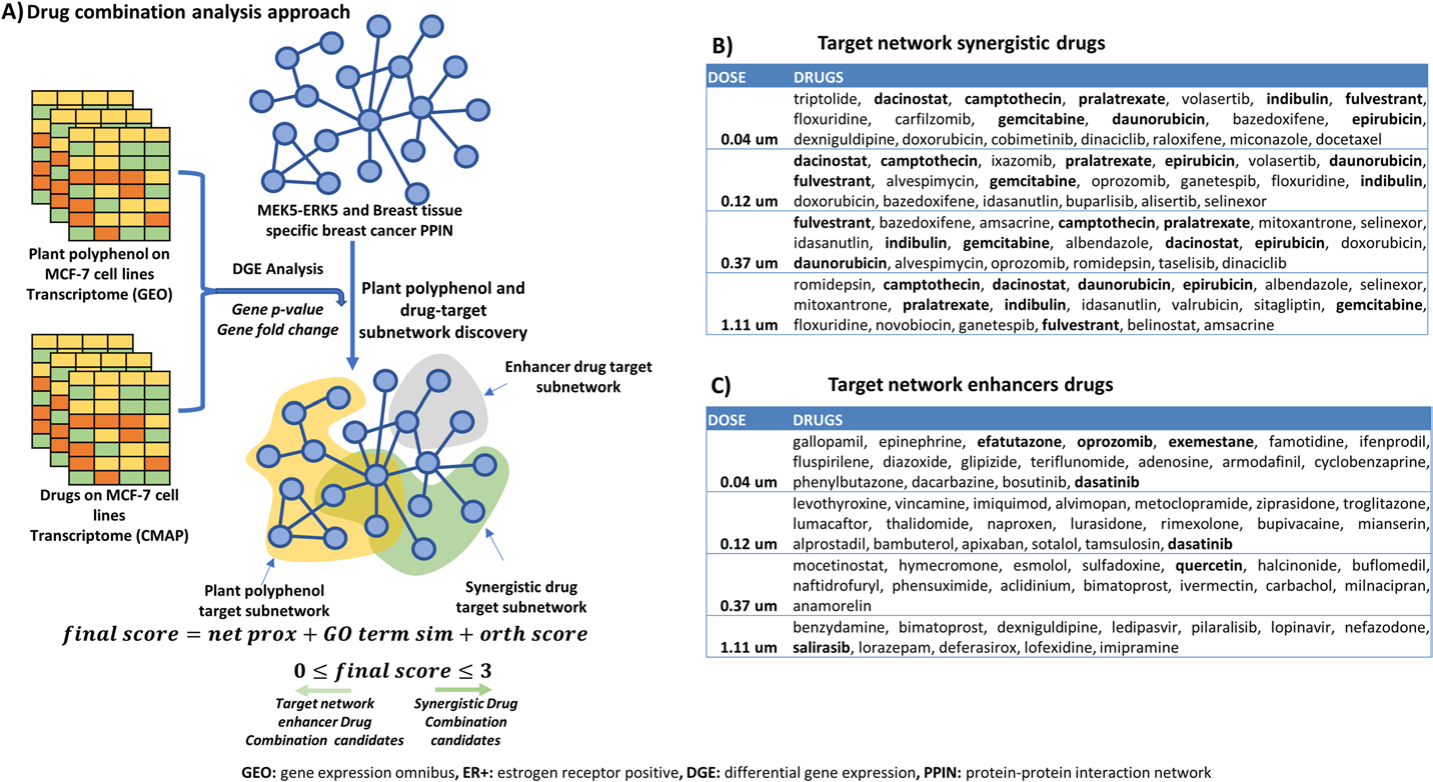
Identificantion of Genistein target subnetwork synergistics and enhancers in the MEK5/ERK5 pathway in breast cancer. **A** A summary of the analysis strategy employed for this analysis showing the key steps from drug data acquisition and processing to candidate drug identification. **B** A plot showing the scores from potential synergistic and **C**) network enhancer drugs obtained by using network proximity score + GO Term similarity (Closest_Score + GOSIM) and network proximity score + GO term similarity + orthogonality score (Final Score) under different doses

For the target network enhancer group of drugs, we found no common drug across all dosages. This category of drugs consisted of drugs indicated for different diseases with a few used as antineoplastic drugs in cancer (Fig. 5B and C). For instance, gallopamil is indicated for abnormal heart rhythms, levothyroxine is the synthetic thyroxine hormone indicated when normal thyroxine levels are low, and hymecromone is an antispasmodic and choleretic agent, while efatutazone, oprozomib, exemestane, and dasatinib, among others are some of the known anticancer drugs identified in this category.

In general, drugs prioritized as synergistic by this approach were mainly antineoplastic drugs. While there were a few antineoplastic drugs in the target network enhancer list of drugs, other classes of drugs such as analgesic drugs (naproxen), antiemetic drugs (metoclopramide), antiviral drugs (ledipasvir), antihypertensive drugs (esmolol) and antacid drugs (famotidine) were also predicted (Fig. 5C and Extended Data Supplementary Tables 7 and 8). In addition, since the majority of the drugs used in this study had approvals from the Food and Drug Administration (FDA) for clinical use, their subsequent investigation as network synergistic or enhancer combinations with plant polyphenols can allow for their faster translation into clinical use.

## Discussion

In the current work, we propose an improved computational network pharmacology pipeline for drug candidate prioritization and drug combination simulation on a contextualized signaling network by using the human protein–protein interaction network as a scaffold. We used plant polyphenol transcriptome datasets in this study due to an increased research interest in their anti-cancer capabilities. The MEK5/ERK5 signalling pathway, which is used as the template, is frequently dysregulated in breast cancer as well as most cancers [60]. Thus, we first showed that the chosen template network was relevant for studying the MEK5/ERK5 signalling pathway, in addition to the cancer-related biological roles associated with this pathway (Fig. 3 and Supplementary Fig. 2). Subsequently, we reconstructed target subnetworks for both the plant polyphenols and drugs (Extended Data Supplementary Table 2). These subnetworks had topological features characteristic of cancer targeted subnetworks, as attested by findings from network degree and betweenness centrality, and thus were instrumental in our network-based analyses (Extended Data Supplementary Table 1).

MEK5/ERK5 has been previously linked with core carcinogenic processes such as avoidance of immune system clearance, enabling replicative immortality, promoting tumor inflammation, genome instability, invasion, metastasis and angiogenesis, and deregulating cellular energetics [20, 60]. We found that the reconstructed MEK5/ERK5 specific subnetwork was enriched with biological processes such as the’MAPK cascade’, ‘cell cycle’, and ‘G2/M transition’, among others (Fig. 3A-C and Extended Data Supplementary Tables 3 and 5). This allowed us to conclude that the reconstructed template network could be used for network-based drug screening analysis. Plant polyphenol prioritization analysis identified Genistein 10µM among all the other compounds and dosages. Indeed, the superiority of Genistein has been clinically proven albeit in postmenopausal women with breast cancer [61]. In addition, the proposed pipeline prioritized drugs with biologically favourable mechanisms of action as potential combination candidates. For instance, dacinostat [62], camptothecin [55], gemcitabine [57], daunorubicin [58], epirubicin [59], fulvestrant [52], and pralatrexate [53] which have either shown good outcome in preclinical studies or are currently approved for clinical use in the management of breast cancer [63] were prioritized as target network synergistic drug combination candidates with genistein 10µM in this study. This finding suggested that these drugs possess similar molecular targets with Genistein. In the literature, genistein has been proposed to modulate the cancer cell cycle, response to growth factors and apoptosis [64]. Incidentally, these are some of the biological functions enriched in the MEK5/ERK5 signalling pathway.

In principle, prioritizing drug combinations with just similar target networks might obscure the holistic thesis in network pharmacology. Thus, this study also highlighted drugs whose target networks were dissimilar to those of plant polyphenols. We regarded these as drugs that when jointly administered would increase the therapeutic spectrum of the treatment regimen. To ensure that they do not induce an undesired response, we applied the same criteria as in the case of synergistic drugs using drug orthogonality as we explain next.

A drug treatment induces a directional change in the expression patterns of the target genes or proteins, i.e., while some genes will increase in expression others will decrease. For a given therapeutic regimen, the objective is to reverse the expression patterns induced by the disease state. Mathematically, the overall influence of the treatment can thus be summarised by computing the corresponding orthogonality. Computing a drug’s orthogonality score, hereby the proposed improvement to the computational network pharmacology approach, is not new in drug target screening. The *signatureSearch* R package implements drug screening on the L1000 CMAP dataset using CMAP, LINCS, and correlation-based techniques, which rely on the directional transformation of the target genes, to identify potential drug candidates [11]. The current study leverages the holistic nature of network-based drug screening pipelines and implements an extra layer for filtering potential drug candidates based on their orthogonality scores from the expression patterns of drug target genes. Indeed, we observed a change in the ranking of both prioritized drugs and potential drug combination candidates using this new screening approach (Fig. 4B and B). Mechanistically, it is more appropriate to expose cancerous cells to treatments that would reverse the expression patterns of disease cause-causing genes, as we considered in this study for synergistic drug combinations. However, this was not implemented before in current network pharmacology-based methods in the literature [4–7].

This study is limited by several factors. The datasets used were generated by different laboratories, and platforms – all of which have their own internal biases that we did not control for during the analysis. The use of cell line-derived drug signature on gene expression has been under criticism owing to the partial mirroring of real-world molecular changes in human patients. Thus, as omics-based drug screening technology develops, integrating data from more reliable model systems into this pipeline will improve the accuracy of network-based drug prioritization and combination simulations. While transcriptome data provide an accessible genome-wide window to the cellular state, they are still limited by precision as not all transcribed genes are translated into proteins. Importantly, laboratory -based validation of the drug combination predictions from this pipeline in future studies will be important to fine-tune the proposed pipeline. In addition, future computational pipelines in this domain should capture drug side effects in both the prioritization and combination simulations to provide a more practical result.

## Conclusion

We propose a flexible computational pipeline to simulate drug prioritization and combinations amenable to different OMIC data, such as transcriptome or proteome data. This pipeline can perform interpretable drug prioritization and combination simulations using a combination of network proximity, GO term enrichment semantic similarity and drug effect orthogonality. The proposed pipeline was able to prioritize Genistein (10µM) and a set of potential drug combinations with strong biological and supporting evidence in the literature.

### Supplementary Information

**Additional file 1: Supplementary Fig. 1.** PCA plots showing a separation between controls from plant polyphenol A) Genistein 3µM, B) Genistein 10µM, C) Ferrulic acid, D) Resveratrol 150mM, E) Resveratrol 250mM, F) Apigenin treated MCF-7 cell line and G) ER + breast cancer respectively. The first two principal components (PC1 and PC2) were used to visualize grouping patterns in each case.

**Additional file 2: Supplementary Fig. 2.** Enrichment analysis results. A-D) Expression patterns of genes in Apigenin 10uM, Genistein 3uM, Genistein 10uM and Reseveratrol 250mM versus ER + breast cancer on MAPK pathway.

**Additional file 3: Supplementary Table 1.** List of genes associated with the MEK5/ERK5 signalling pathway and enriched in human breast tissue. These genes were obtained after merging evidence from the DisGeNet and the Human Proteome Atlas databases. **Supplementary Table 2.** Topology analysis of plant polyphenols target MEK5/ERK5 pathway networks. Disease association information was extracted from the DisGeNet database with only genes curated in breast cancer selected. Nodes with low betweenness centrality scores (betweenness = 0) were removed. **Supplementary Table 3.** Pathway enrichment analysis results based on KEGG, GO, and DisGeNet showing the enriched terms in the nodes of subnetworks of each plant polyphenol. **Supplementary Table 4.** Results of drug-target subnetwork topological analysis showing the number of proteins (nodes) and their interactions (edges) at different doses. **Supplementary Table 5.** List of enriched pathways in the set of common genes between plant polyphenol and breast cancer target subnetworks. **Supplementary Table 6.** Differences between the network topology and overall average network topology for each plant polyphenol target network. **Supplementary Table 7.** List of drugs prioritized as combination candidates with target network synergistic effects. Different dosages of each drug were used in the simulation and for each dose the first 10 best scored drugs are provided. The network scores as well as the number of genes in common between drug-deregulated genes and the background network are shown. **Supplementary Table 8.** List of drugs prioritized as combination candidates with target network enhancer effects. Different dosages of each drug were used in the simulation and for each dose, the first 10 best scored drugs are provided. The network scores as well as the number of genes in common between drug-deregulated genes and the background network are shown.

## Data Availability

All codes used in this study can be accessed via our GitHub page at https://github.com/SysBioGTU/NetworkPharmacology. All data used in this study are publicly available in the Gene Expression Omnibus and other sources. The data sources have been clearly referenced as mentioned.

## Abbreviations

MAPK: Mitogen activated protein kinase
ERK5: Extracellular signal regulated kinase 5
MEK5: MAPK/ERK kinase 5
GO: Gene ontology
BP: Biological process
MF: Molecular function
µM: Micromolar
mM: Millimolar
GEO: Gene Expression Omnibus
PCA: Principal component analysis
ER +: Estrogen receptor positive
LA: Luminal A
|log2FC|> 1.2: Absolute log2 Fold change
DGIdb: Drug-gene interaction database
HIPIE: Human integrated protein–protein interaction reference
FDR: False discovery rate
DisGeNet: Disease-gene network
KEGG: Kyoto encyclopaedia of genes and genomes
PPIN: Protein–protein interaction network
CMAP: Connectivity map
FDA: Food and Drug Administration
