# Supplementary material for: A network-based drug prioritization and combination analysis for the MEK5/ERK5 pathway in breast cancer
Source: BioData Min. 2024 Feb 21;17:5. doi: 10.1186/s13040-024-00357-1 (PMC10880212; doi:10.1186/s13040-024-00357-1)
Supplement: Supplementary file 1 — Additional file 1: Supplementary Fig. 1. PCA plots showing a separation between controls from plant polyphenol A) Genistein 3µM, B) Genistein 10µM, C) Ferrulic acid, D) Resveratrol 150mM, E) Resveratrol 250mM, F) Apigenin treated MCF-7 cell line and G) ER + breast cancer respectively. The first two principal components (PC1 and PC2) were used to visualize grouping patterns in each case. [file 13040_2024_357_MOESM1_ESM.docx]

**A**


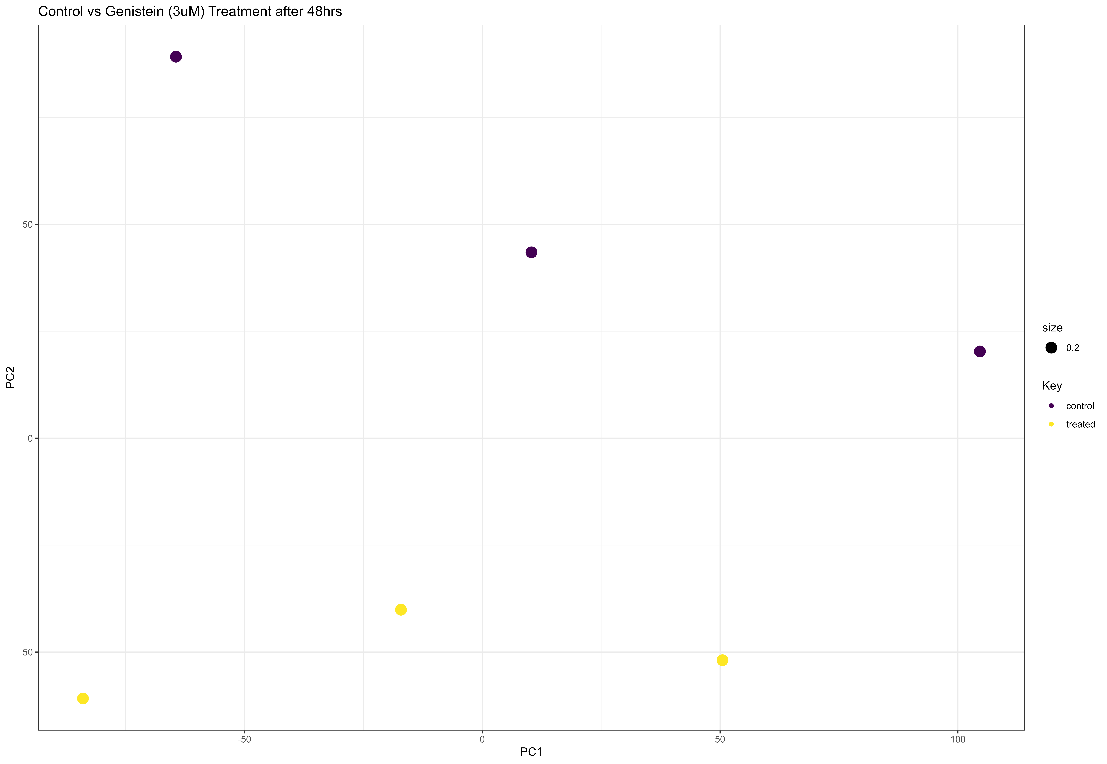


**B**


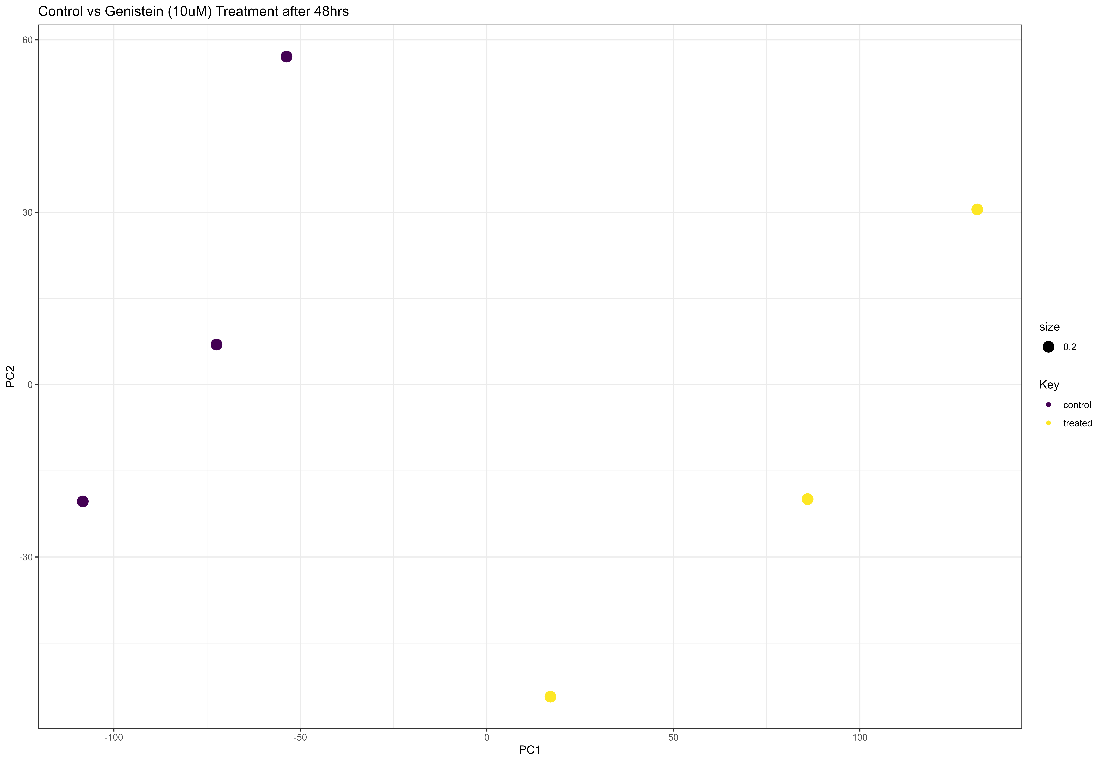


**C**


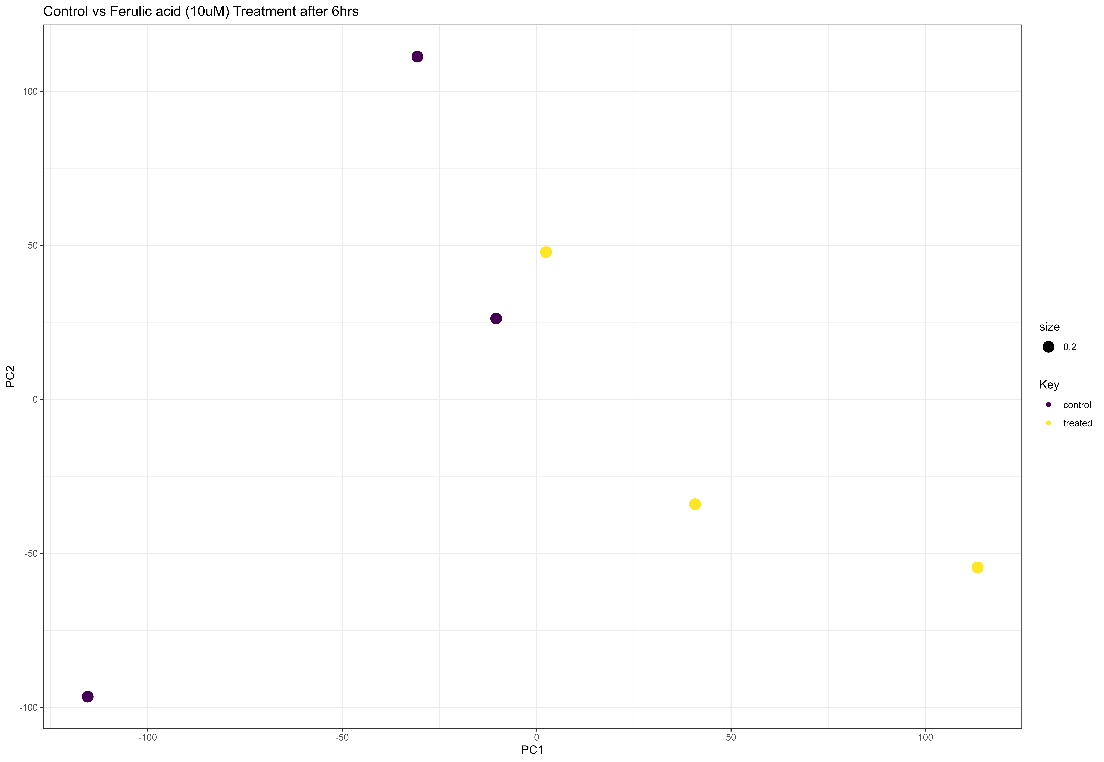


**D**


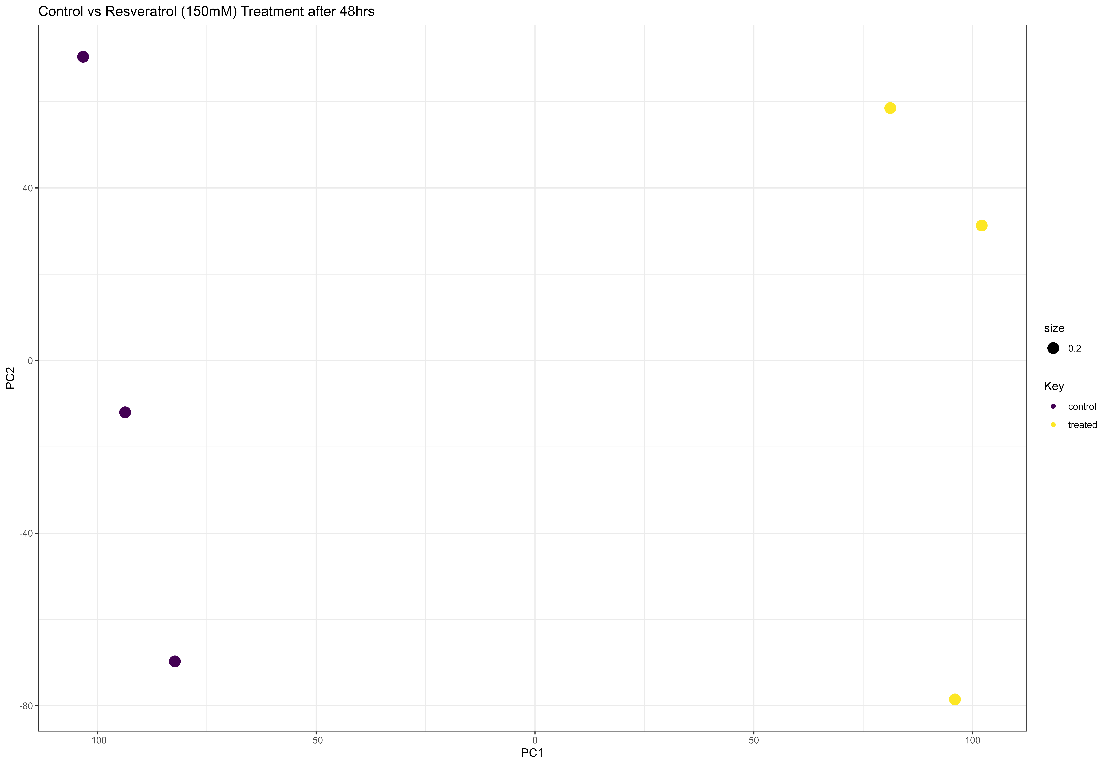


**E**


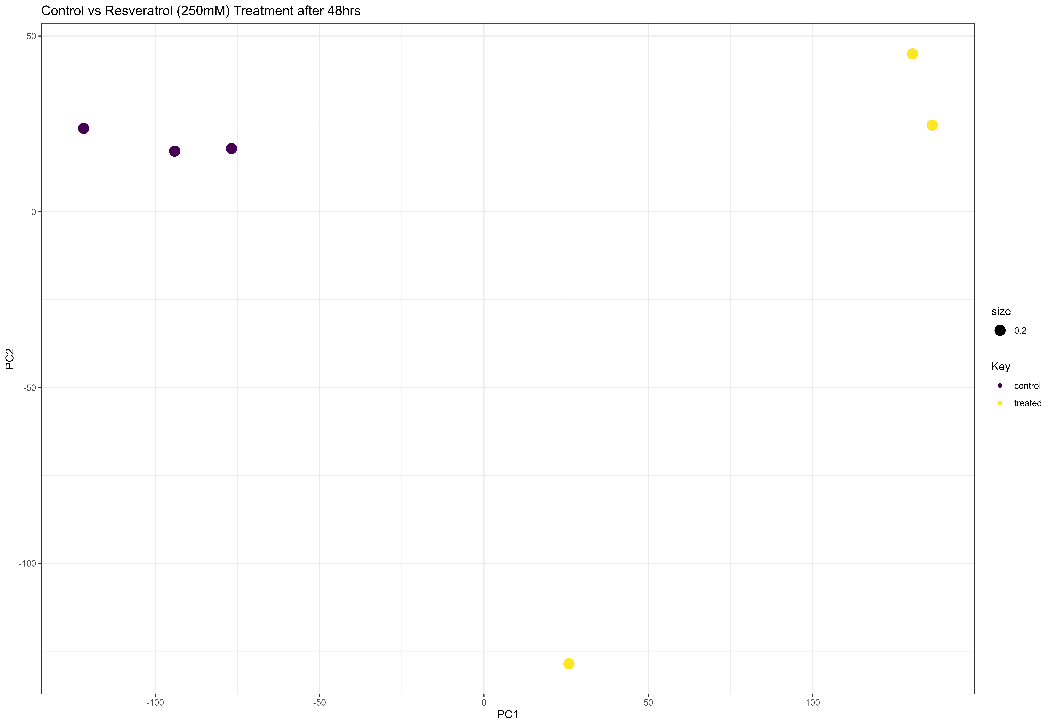


**F**


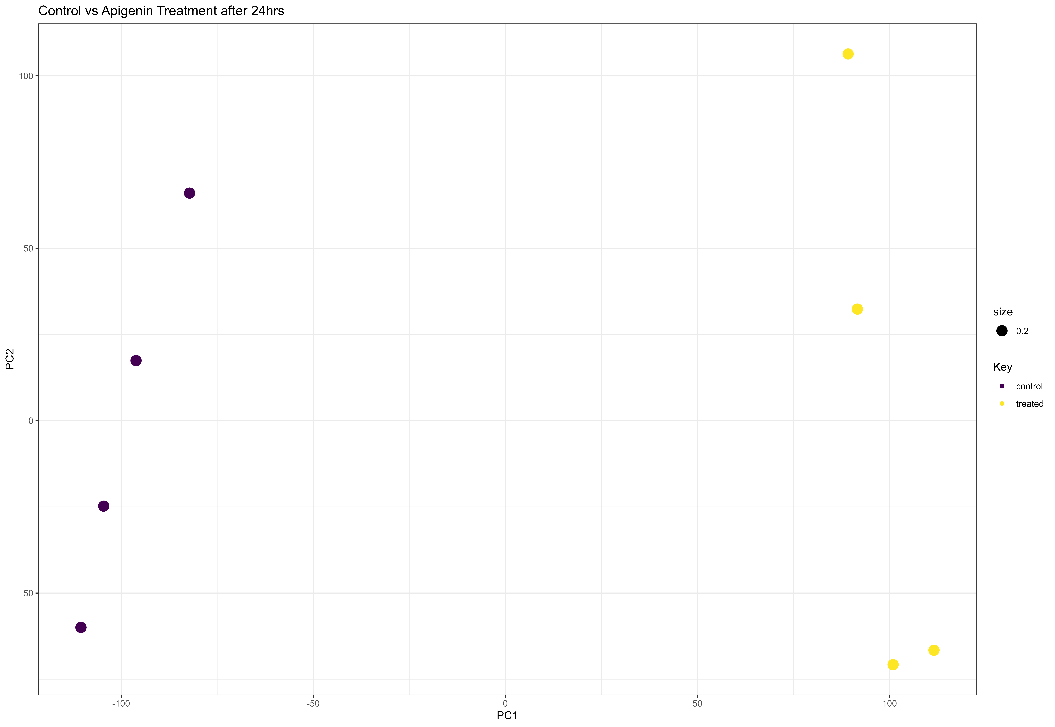


**G**


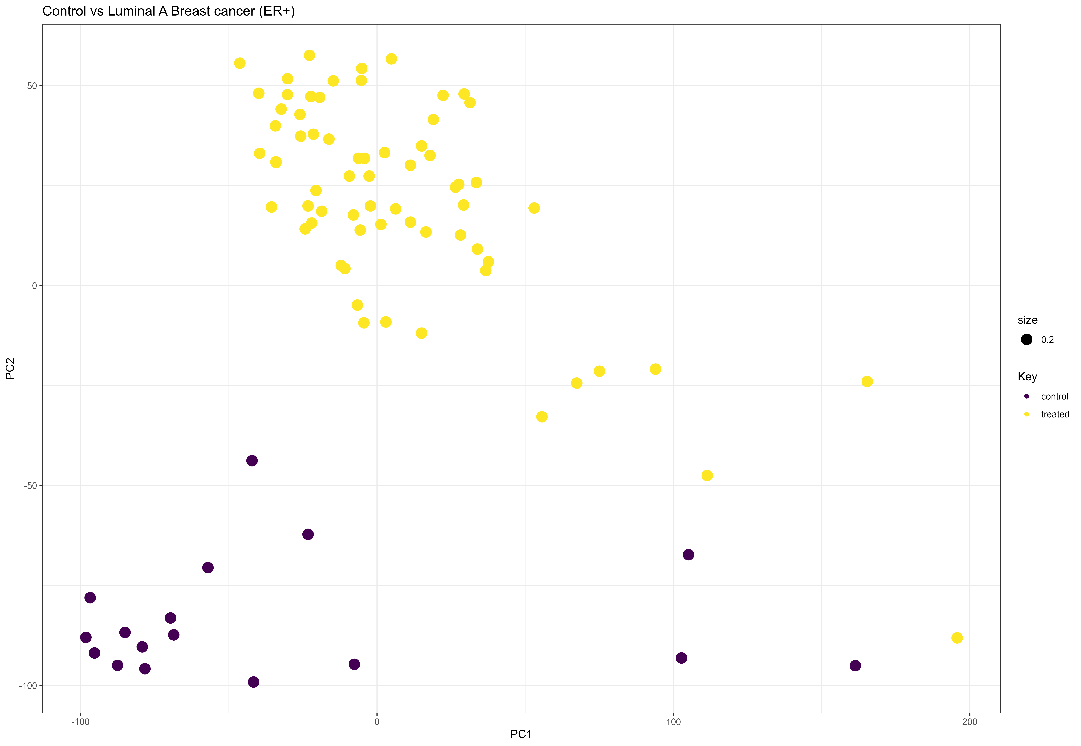


**Supplementary Figure 1**: PCA plots showing a separation between controls from plant polyphenol A) Genistein 3µM, B) Genistein 10µM, C) Ferrulic acid, D) Resveratrol 150mM, E) Resveratrol 250mM, F) Apigenin treated MCF-7 cell line and G) ER+ breast cancer respectively. The first two principal components (PC1 and PC2) were used to visualize grouping patterns in each case.
