# Supplementary material for: A network-based drug prioritization and combination analysis for the MEK5/ERK5 pathway in breast cancer
Source: BioData Min. 2024 Feb 21;17:5. doi: 10.1186/s13040-024-00357-1 (PMC10880212; doi:10.1186/s13040-024-00357-1)
Supplement: Supplementary file 2 — Additional file 2: Supplementary Fig. 2. Enrichment analysis results. A-D) Expression patterns of genes in Apigenin 10uM, Genistein 3uM, Genistein 10uM and Reseveratrol 250mM versus ER + breast cancer on MAPK pathway. [file 13040_2024_357_MOESM2_ESM.docx]

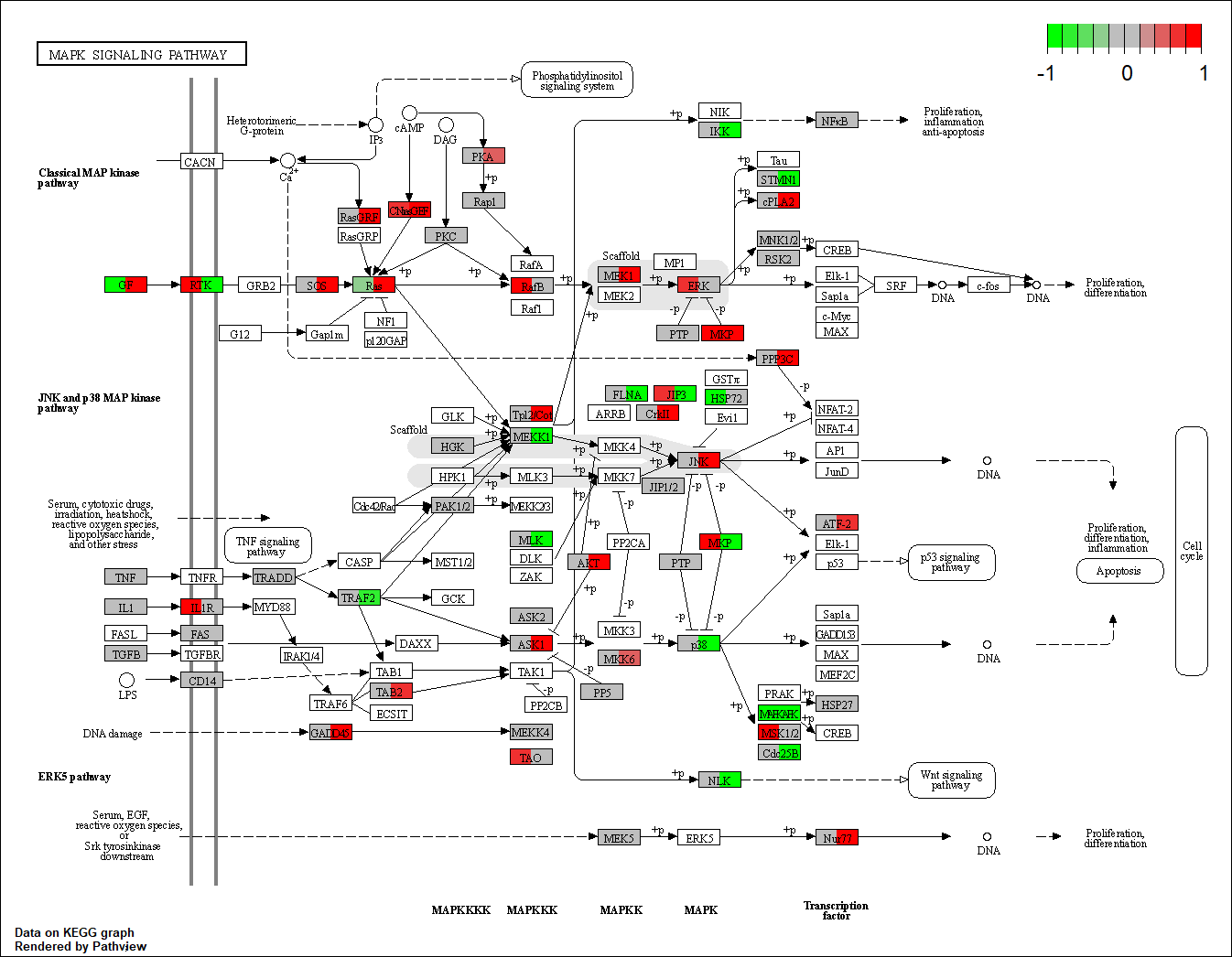


A)


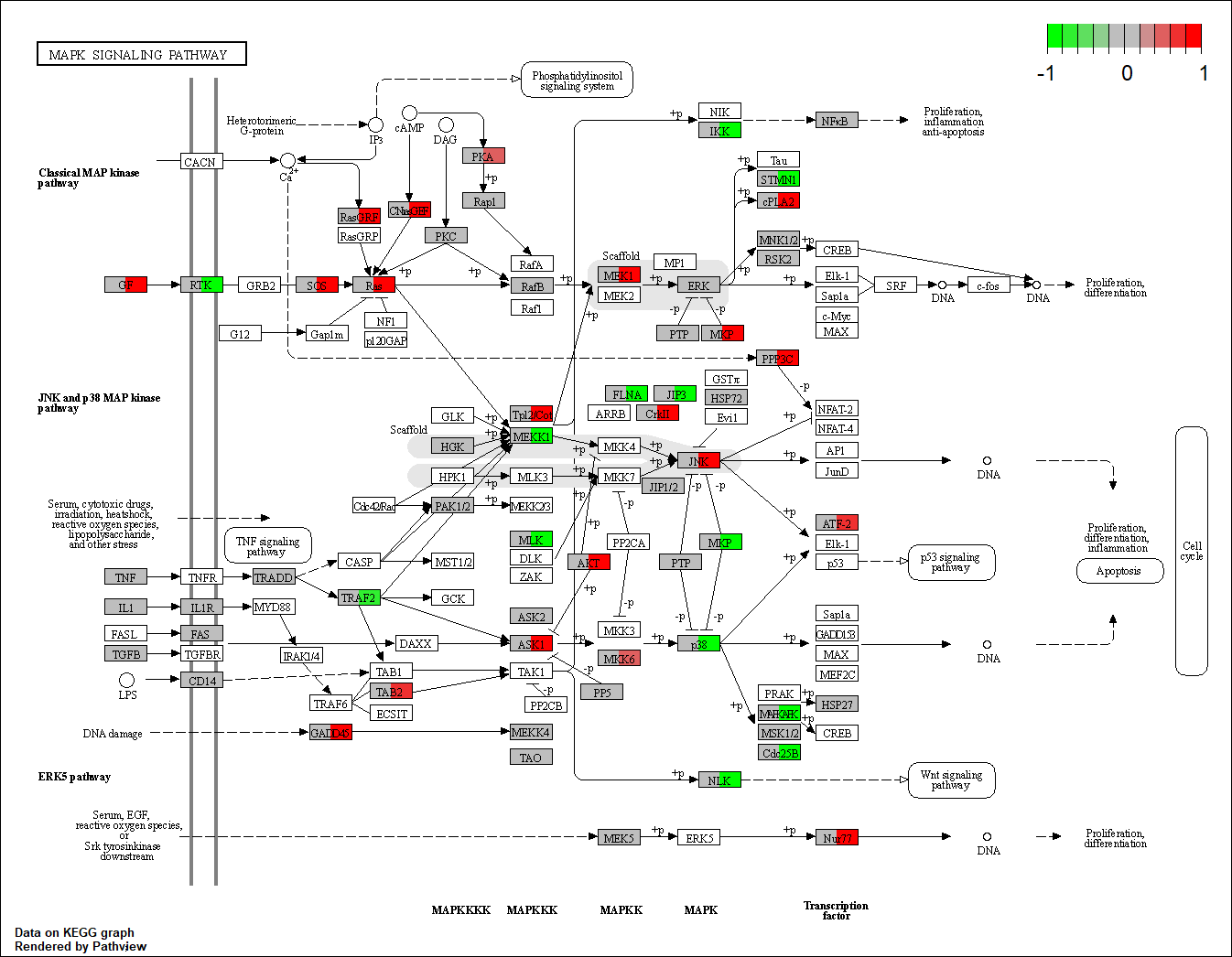


B)


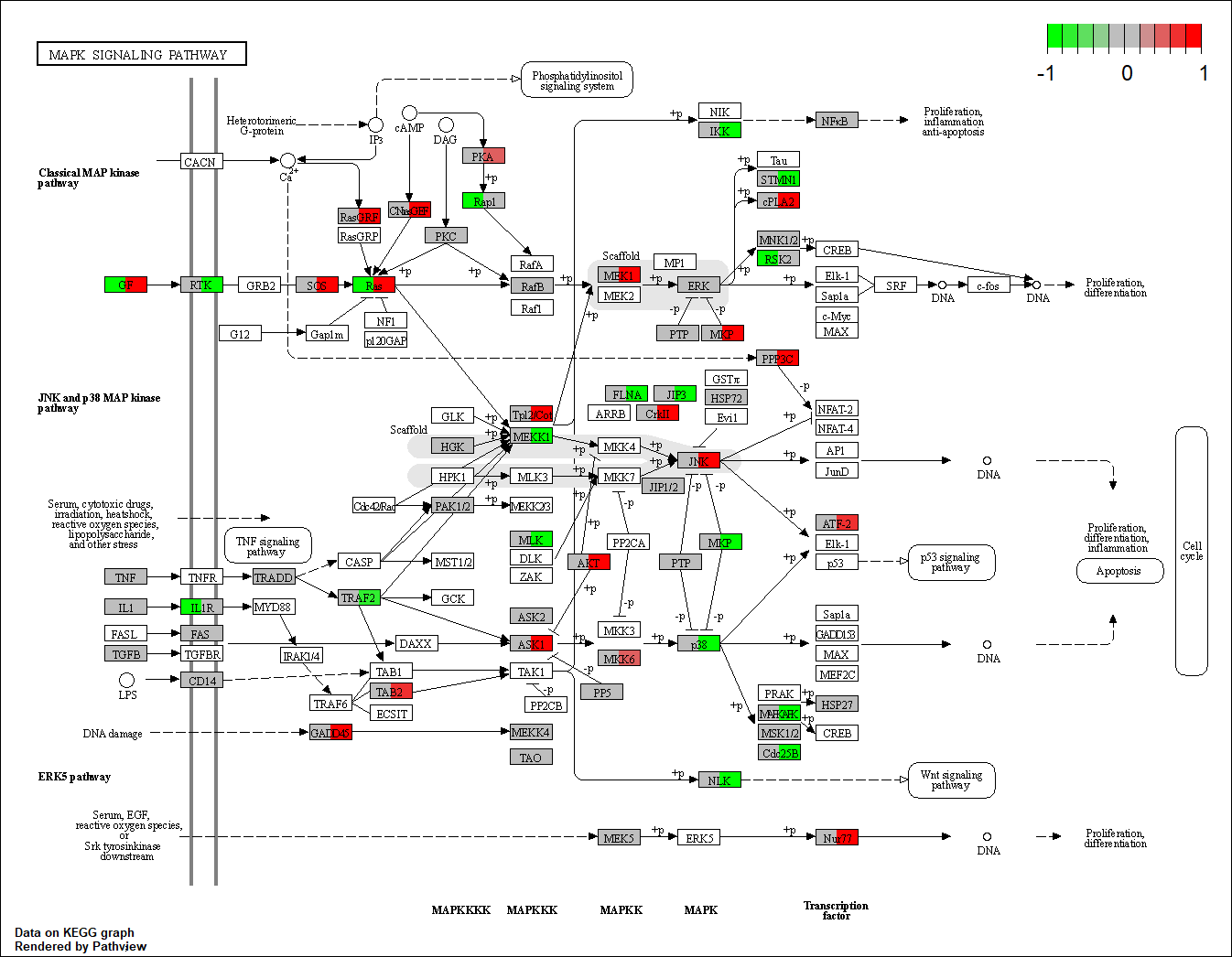


C)


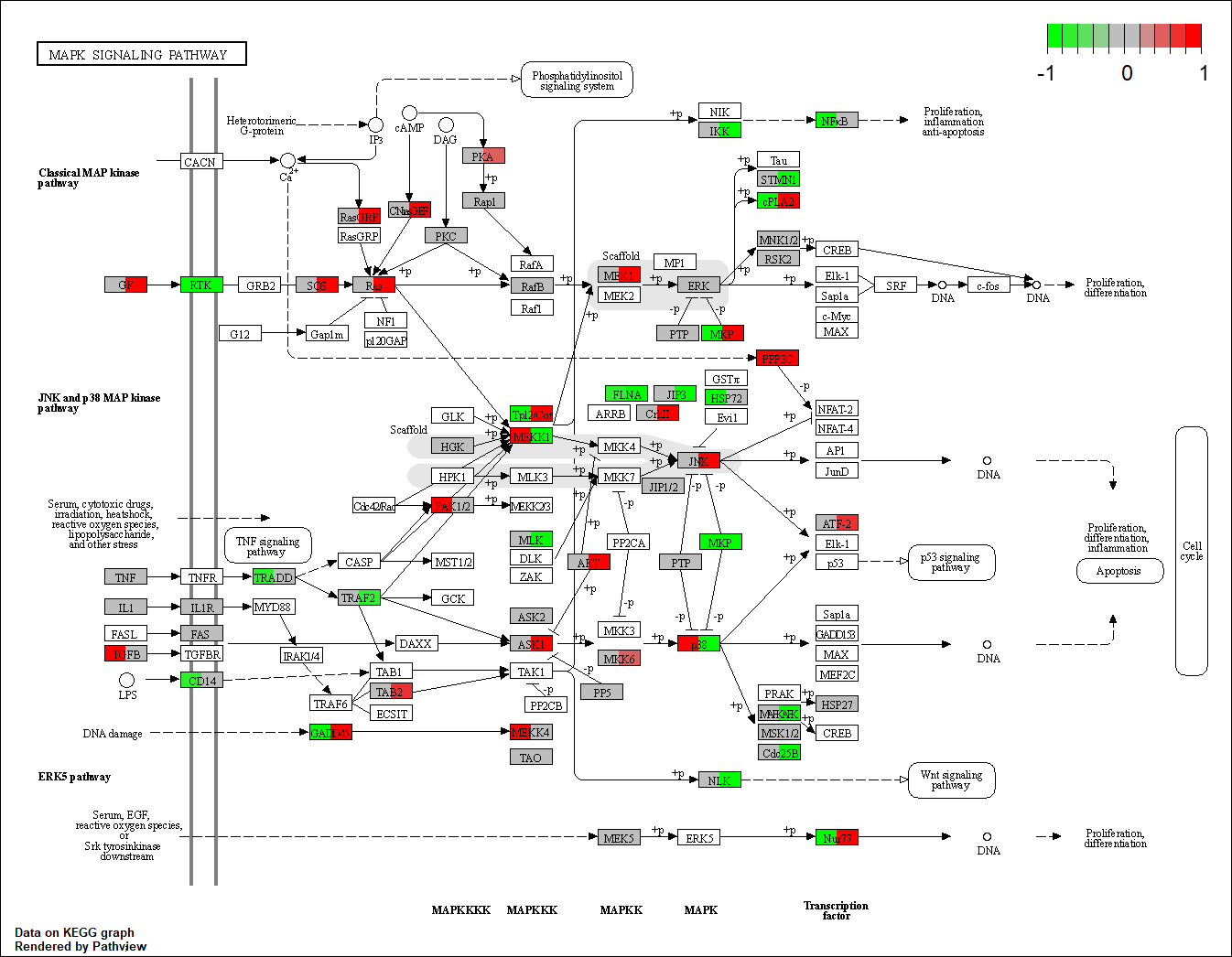


D)

**Supplementary Figure 2**: Enrichment analysis results. A-D) Expression patterns of genes in Apigenin 10uM, Genistein 3uM, Genistein 10uM and Reseveratrol 250mM versus ER+ breast cancer on MAPK pathway.
